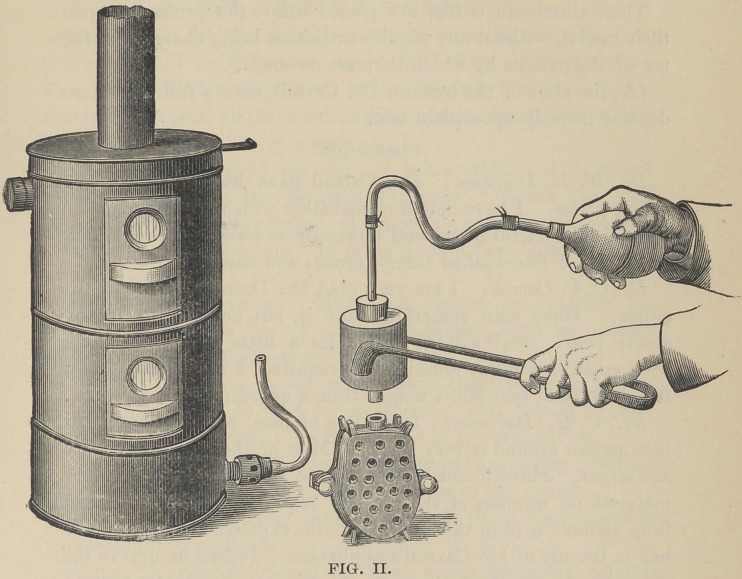# Aluminum Cast Dentures

**Published:** 1888-06

**Authors:** C. C. Carroll

**Affiliations:** Meadville, Pa.


### Aluminum Cast Dentures.

BY DR. C. C. CARROLL, OF MEADVILLE, PA.

[Given as a clinic at the Michigan State Dental Association held at Ann Arbor, March 20, 1888.]

Aluminum has long been regarded as possessing many properties peculiarly fitting it as a base for dental plates, such as lightness, stiffness, conductivity and strength ; yet it has certain peculiarities differing from other metals that have precluded its general use in the mechanic arts, and especially in dentistry until quite recently. Two objections to its use as a swaged plate have existed. First, our inability to solder it; and second, when swaged dentures with rubber attachments have been made, it has been found in many cases to disintegrate or become porous by the action of the fluids of the mouth. As the result of a long conducted series of experiments, it has been discovered that the disintegration was caused by iron and other impurities found in the aluminum of commerce, which was used for swaged plates. Efforts have been made during the past quarter of a century to cast it into dentures, but its low specific gravity of only two and five-tenths, and its great contraction of a line to the inch in cooling, were the difficulties in the way of a cast denture. All these difficulties are now overcome in our prepared aluminum, which is first made chemically pure to prevent disintegration, then alloyed with a small per cent. of noble metals that expand in cooling and thus compensate the contraction of the aluminum, reducing the contraction to the one-tenth part of a line, or the one one-hundred-and-twentieth part of an inch, practically nil, enabling us to cast directly upon the teeth without a fracture. The difficulty of making a sharp cast of aluminum by virtue of its low specific gravity is overcome by the use of our pneumatic crucible, which enables us to force the molten aluminum into every part of the matrix producing a perfect cast of the model.

We take an impression for this aluminum cast work as we would for any other work; then from this impression make a model of plaster-of-Paris, three parts, and of fine sand or marble dust, one part. Now we proceed very much as in rubber work. For temporary base plates we take common paraffine wax and roll it down to about twenty-three standard gold plate gauge.

There are various forms of mounting the common rubber teeth which we used in this aluminum cast work. The simplest of which is to cast a base plate with a flange or undercut for the purpose of attaching the teeth by pink rubber or celluloid. Upon this cast base plate we place wax and get the bite, which we place upon the articulator and mount in the usual manner for rubber work. Then attach the teeth to this aluminum base plate, making an artificial gum of pink rubber or celluloid.

Another form of mounting is to place plain teeth directly upon the temporary wax base plate, the same as in mounting for rubber work, with the exception that you space your teeth slightly to allow for this slight contraction. Along the alveolar border we make an undercut in the wax base plate which undercut is reproduced in the aluminum plate permanently when cast, for the attachment of a gum colored facing of pink rubber or celluloid. We now invest the tooth upon the model in the two-part perforated iron flask very much after the manner of investing for rubber work, Fig. 1. Cut gates from the center part of the base plate to the pouring point of the flask, also pockets from the heel of the base plate into which the air is forced through the matrix in the act of casting. The wax base plate is removed by washing out with hot water, and the flask placed in the upper chamber of the automatic gas (or gasoline) furnace to be dried out preparatory to casting. You will observe that by this method of mounting we intend to cast the aluminum directly upon the teeth, attaching them firmly to the plate.

Gum section teeth can be used as well as plain teeth by exercising care in the method of mounting, taking the precaution of placing a thin slip of paper between the joints before investing.

When the matrix is dry, which will be shown if no moisture appears upon a mouth mirror held over the pouring point, we make the cast by use of an automatic crucible, which is placed in the lower chamber of the furnace and contains the aluminum to be used in casting. The crucible is placed upon the flask, connecting the nipple of the pneumatic crucible with the pouring point of the flask, and by means of a rubber bulb the aluminum is forced into the matrix, making a very sharp and well defined cast which is a perfect counterpart of the model, Fig. II. As

FIG. II.

soon as the piece has cooled, the flask is opened and the denture removed. The piece is then finished up by means of sand paper and pumice stone, using fine crocus for a finer polish. It takes and retains the appearance of the finest polished nickel plate.

All forms of dentures are readily made by means of this aluminum cast work, including crowns, bridges, as well as partial and complete dentures. This system of aluminum cast work is entirely new, simple and complete, and promises to revolutionize the present method of prosthetic dentistry.

While aluminum, by virtue of its extreme lightness, having a specific gravity of 2.5, is peculiarly fitted for upper dentures, it is better to have a heavier metal for lower dentures. For this purpose we make an alloy containing a specific gravity of 7.5 This alloy, Base No. 2, is the solder for Base No. 1, and is used for lower dentures partial or complete. By means of Base No. 2 a piece may be readily soldered and mended.

These aluminum outfits are placed before the profession upon their merits, without any royalty or license being charged for the use of the patents by which they are covered.

(At the close of the lecture, Dr. Carroll cast a full aluminum denture directly upon plain teeth.

DISCUSSION.

*Dr. W. H. Dorrance:* Dr. Carroll gave his method of polishing plates. I have here a composition which is very valuable for polishing metal plates and especially so for aluminum. It is composed of fine-washed emery, crocus and hard oil.

*Dr. C. C. Carroll:* I am very glad Dr. Dorrance spoke of the polish. Have seen something like it, but never gave it any special trial. Shall ask the doctor for a little to try. Having been asked how much aluminum is required, I would say that a full denture of base No. 1 weighs from a quarter to half an ounce.

*Dr. C. H. Harroun:* This plate of base No. 1, which has been passed around is very small. Most of the plates we make are larger. Have found one must use nearly an ounce of the material on account of the surplus required in casting. I can fully endorse it from the several months experience which I have had in the use of Dr. Carroll's appliances. It feels natural in the mouth being the best substitute for the natural denture that I have found. My patients say so. I have used it in some of the most difficult cases in my practice, and have had a perfect success in every case.

*Dr. Carroll:* If so much material is used, it is not wasted. It can be re-cast time and again, thus utilizing all the scraps.

*Dr. C. H. Harroun:* I use kerosene oil for separating when investing.

*Dr. W. H. Dorrance:* I can recommend to the Doctor something much cheaper. It does not cost near so much. It is water.

*Dr. Carroll:* I use pulverized soapstone for separating the investments. Aluminum does not oxidize Molten aluminum is not necessarily fluid aluminum. It has, however a flux, bicarbonate of soda that will cause it to flow readily, this should be used in re-melting scraps.

*Dr. J. Taft:* This metal is an alloy. Will both metals oxidize alike? So that if in re-casting to save the waste the proportion should be destroyed, would the alloy not deteriorate? This is a question important in considering the use of this material. Again, might it not oxodize in some conditions of the atmosphere? Ozone is very active.

*Dr. Carroll:* The $3\frac{1}{2}$ per cent. of noble metal is lost except so far as to cause the required expansion. While the gold case of my watch was effected at the seashore by the ozone, the aluminum plates were not. It is an unoxidizable metal in the atmosphere.

*Dr. W. H. Dorrance:* Must the doctor not caution his patients against the use of certain medicines, such as contain hydrochloric acid, for instance?

*Dr. Carroll:* Just as all metals have there solvents, hydrochloric acid is the solvent of aluminum. But this is so only in its full strength, which we do not find in the oral cavity.

*Dr. Parker:* Have found amalgam to have a strong galvanic action on coming in contact with aluminum in the mouth.

*Dr. Carroll:* That can occur only when there is an excess of mercury in the amalgam.

When Dr. Carroll opened the flask which had been allowed to cool he said, to the question as to what effect common salt has upon aluminum plates, that of all his patients no doubt eating salt daily, there had been no effects. He went on to say: There is sufficient specific gravity in base No. 2 so that it does not require to be forced into the flask by pneumatic pressure like base No. 1. Do not melt the aluminum in an iron crucible, on account of its affininity for oxide of iron. Use bicarbonate of soda as a flux when melting.

*Dr. C. H. Harroun:* Will it do to melt the aluminum in one of our common crucibles?

*Dr. Carroll:* Those crucibles are made of clay, which is silicate of aluminum. If you wish to use such a one, dilute it with whiting as you do when you use it for melting gold. This crucible is made of plumbago. If the nipple should come out of the crucible at any time on account of some mistake in handling it, a cement may be made after this formula: Silicate of soda and pulverized soap-stone; add a little water and rub into a paste. Having applied it, put the piece upon the furnace until it becomes dry. Later, when it is heated, it will become very hard. Some one asked me how one may know when to make the cast. Place the nugget of aluminum into the crucible, and when it melts down you may know it is ready.

## Figures and Tables

**Fig. I. f1:**
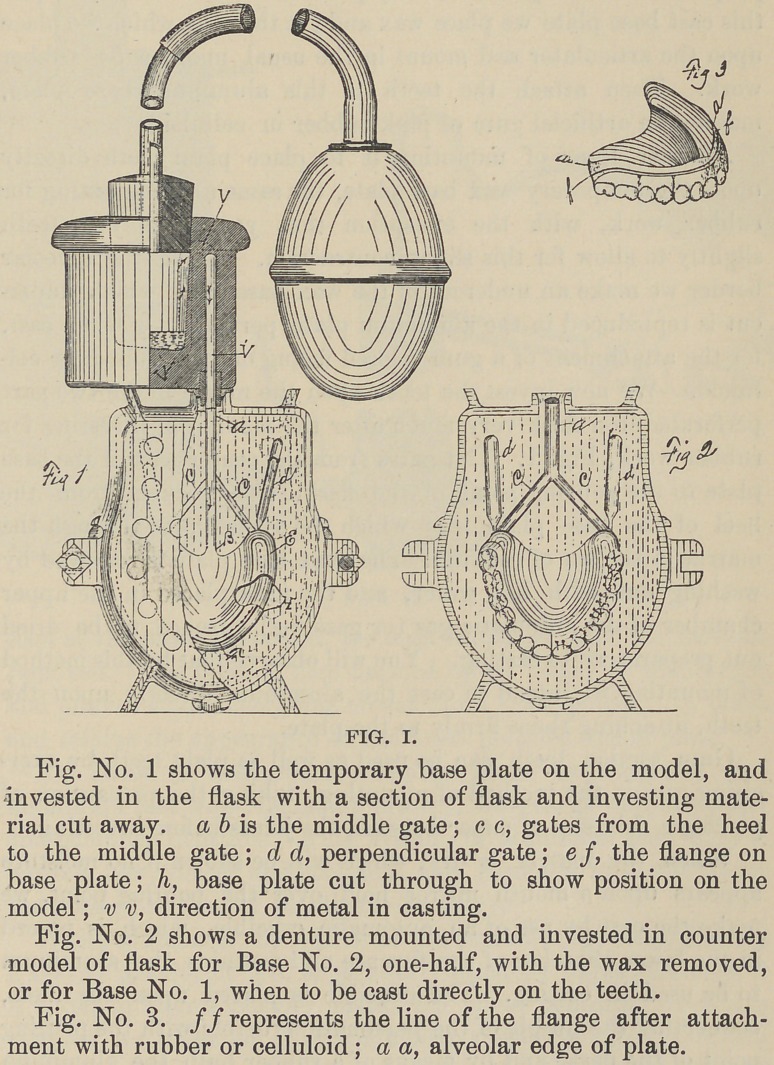


**Fig. II. f2:**